# Animal Models of Cancer-Related Pain: Current Perspectives in Translation

**DOI:** 10.3389/fphar.2020.610894

**Published:** 2020-11-26

**Authors:** Jorge B. Pineda-Farias, Jami L. Saloman, Nicole N. Scheff

**Affiliations:** ^1^ Department of Neurobiology, University of Pittsburgh School of Medicine, Pittsburgh, PA, United States; ^2^ Division of Gastroenterology, Hepatology, and Nutrition, Department of Medicine, University of Pittsburgh School of Medicine, Pittsburgh, PA, United States; ^3^ Hillman Cancer Center, University of Pittsburgh Medicine Center, Pittsburgh, PA, United States

**Keywords:** cancer pain, animal model, cancer treatment, nociception, behavior

## Abstract

The incidence of pain in cancer patients during diagnosis and treatment is exceedingly high. Although advances in cancer detection and therapy have improved patient prognosis, cancer and its treatment-associated pain have gained clinical prominence. The biological mechanisms involved in cancer-related pain are multifactorial; different processes for pain may be responsible depending on the type and anatomic location of cancer. Animal models of cancer-related pain have provided mechanistic insights into the development and process of pain under a dynamic molecular environment. However, while cancer-evoked nociceptive responses in animals reflect some of the patients’ symptoms, the current models have failed to address the complexity of interactions within the natural disease state. Although there has been a recent convergence of the investigation of carcinogenesis and pain neurobiology, identification of new targets for novel therapies to treat cancer-related pain requires standardization of methodologies within the cancer pain field as well as across disciplines. Limited success of translation from preclinical studies to the clinic may be due to our poor understanding of the crosstalk between cancer cells and their microenvironment (e.g., sensory neurons, infiltrating immune cells, stromal cells etc.). This relatively new line of inquiry also highlights the broader limitations in translatability and interpretation of basic cancer pain research. The goal of this review is to summarize recent findings in cancer pain based on preclinical animal models, discuss the translational benefit of these discoveries, and propose considerations for future translational models of cancer pain.

## Introduction

Cancer-related pain can occur at any time during the evolution of the disease (Caraceni and Shkodra, 2019). Many patients present with pain as the first sign of cancer, and 30–50% of all cancer patients will experience moderate to severe pain; frequency and intensity of pain can increase with cancer progression (Mercadante, 1997; Mercadante and Arcuri, 1998). Despite significant advances in cancer treatment as well as early detection, cancer-related pain treatment strategies remain limited. Opioids remain the current therapeutic regimen for cancer-related pain, based on the World Health Organization (WHO) analgesic ladder (Nersesyan and Slavin, 2007), despite debilitating side effects and inadequate efficacy (Mandala et al., 2006). The limited neurobiological understanding in analgesia pharmacology has restricted novel therapeutic development; cancer pain treatments have relied largely on scientific advancements in other pain conditions.

Animal models of cancer-related pain have provided mechanistic insights into how cancer pain is generated and progresses under a constantly changing molecular architecture. *Cancer* pain is thought to result from processes involving crosstalk between neoplastic cells, the host’s immune system, and peripheral and central nervous systems (Jimenez Andrade and Mantyh, 2010; Lozano-Ondoua et al., 2013; Schmidt, 2014). The field of cancer pain is just beginning to apply nociceptive behavioral assays to established rodent cancer models to reflect the symptoms experienced by patients. The goal of this review is to assess translational ability of the current animal models of cancer pain to the clinical presentation and provide a reference and direction for researchers studying cancer pain.

## Clinical Assessment of Cancer Pain

Assessment of cancer-related pain is broken down into that arising directly from the tumor (85%), pain due to disease progression (9%), pain as a side effect of treatment (17%) (e.g., chemotherapy, surgical resection), and pain from other causes not related to malignancy (Grond et al., 1996). *Cancer* can affect any type of tissue, including viscera, bone, soft and nervous tissue. Pain can arise from the original site of the cancer (e.g., pancreas, head and neck) or from distant sites (e.g., bone), where common cancers metastasize (Coleman, 2006). *Cancer* patients often experience pain at multiple sites. Focal pain is experienced at a single site, usually in the region of the underlying lesion. Referred pain is denoted as progressive pain in a site lacking focal pathology (Caraceni and Portenoy, 1999; Portenoy and Ahmed, 2018). Physiological mechanisms in focal cancer pain are broadly described as nociceptive, inflammatory, or neuropathic (Falk and Dickenson, 2014). Nociceptive pain can be further classified into somatic and visceral. Somatic nociceptive pain is usually well localized and described as sharp, aching, throbbing, or pressure-like. When caused by obstruction, visceral nociceptive pain is often described as gnawing or crampy; when caused by involvement of organ capsules or mesentery, visceral pain may be aching, sharp, or throbbing. Neuropathic pain, defined as pain caused by a lesion or damage to the nervous system, is present in about 39% of patients including those with mixed pain (i.e., including both a nociceptive and neuropathic component) (Bennett et al., 2012); it is described as burning, tingling, or shock-like (lancinating) (Caraceni and Portenoy, 1999; Portenoy and Ahmed, 2018).

Due to disease progression and often resulting tissue damage, the temporal variation of cancer-related pain is classified as acute, chronic, or intermittent. Acute pain is defined by recent onset and brevity. Chronic cancer pain persists for three or more months, often increases with disease progression, and may regress with tumor shrinkage. Chronic pain may be associated with affective disturbances (e.g., anxiety, depression) as well as vegetative symptoms (e.g., anorexia, sleep disturbances) (Bennett et al., 2019). In the cancer population, a brief increase of intense pain in the presence of cancer pain successfully managed with opioid drugs is common and has been defined as “breakthrough” pain; it is estimated that more than one in two patients with cancer pain will also experience breakthrough pain (Portenoy and Hagen, 1990; Deandrea et al., 2014).

Advances in cancer treatment have significantly prolonged survival, and thus the appearance of pain as a sequela is becoming more prominent (Bennett et al., 2012; Liu et al., 2017). *Cancer* treatment-related pain may include bone pain immediately after radiotherapy (Loblaw et al., 2007; Hird et al., 2009) and post-surgical pain due to mastectomy, neck lymph node dissection, laparotomy, and thoracotomy or due to nerve sacrifice during surgery, such as post-thoracotomy pain syndrome (Brown et al., 2014; Liu et al., 2017). Mixed pain is common when caused by cancer treatments (Urch and Dickenson, 2008; Fallon, 2013); however, pain resulting from chemotherapy, termed chemotherapy-induced peripheral neuropathy (CIPN), is the most common and purely neuropathic in nature (Lema et al., 2010). CIPN usually exhibits dose-dependence and distribution through the upper and lower extremities. CIPN can persist for several months to years, even after discontinuation of chemotherapy. Symptoms can include sensory loss, paresthesia, dysesthesia, and pain (Loprinzi et al., 2007; Grisold et al., 2012; Sisignano et al., 2014).

## Animal Models of Cancer-Related Pain

To study cancer pain in the laboratory setting, several animal models have been developed over the last 2 decades to assess pain related to cancers in somatic and visceral tissues as well as cancer treatment related pain. These models seek to reflect the complex pain state observed clinically by measuring mechanical, thermal, and spontaneous pain-related behaviors.

### Cancer-Related Pain Models

The transplantation model is the most popular model in focal and metastatic cancer pain research, and investigators have utilized several permutations. The two broad categories of transplant models are xenograft and allograft (i.e., syngeneic). Xenografts utilize commercially available human tumor cell lines; allografts use tumor cell lines derived from species with the same genetic background (e.g., mouse, rat). A variety of cell lines have been used, all of which have provided insights into the similarities and differences by which different tumors drive cancer pain; a comprehensive list is compiled in Table 1. The major benefits of transplantation models are easy replication, high modeling success rate, and high stability to facilitate the generation of abundant experimental mice. Orthotopic tumors provide a more comprehensive assessment of nociceptive behavior in response to tumor growth within an experimental environment more like the origin site (i.e. anatomical structure and sensory fiber innervation). Immunodeficiency is required for xenograft models, which is an important component when considering the multifaceted cancer pain phenotype. While immune cells are not absent in the cancer microenvironment (Chodroff et al., 2016), the loss of important signaling lymphocytes may lead to clinically irrelevant infiltration and neuro-immune communication.

**TABLE 1:** Transplantation cancer pain models.

*Cancer* type	Cell line	Injection site	References
**Xenograft transplant models**			
Prostate	ACE-1, PC3, PPC-1	Bone, hindpaw	(Halvorson et al., 2005b; King et al., 2008; Thudi et al., 2008; Jimenez-Andrade et al., 2010)
Pancreas	SW 1990, Panc1, AsPc-1, Capan1	Pancreas, bone, sciatic, flank	(Wang et al., 2017; Miura et al., 2018; Han et al., 2020)
Melanoma	WM-127	Hindpaw	(Pickering et al., 2008)
Oral	HSC3, HSC2	Hindpaw, tongue, floor of mouth	(Pickering et al., 2008; Ye et al., 2011; Ye et al., 2012; Chodroff et al., 2016; Lam, 2016; Scheff et al., 2017)
Breast	MDA-MB-231-BO	Bone, mammary pad	(Hiraga et al., 2001; Bloom et al., 2011; Ungard et al., 2014; Zinonos et al., 2014)
**Allograft transplant models**			
Pancreas	PANC02, KPC K4242	Pancreas	(Selvaraj et al., 2017)
Prostate	AT3B-1, AT-3.1, AT3B, MATLyLu	Bone	(King et al., 2008; Jimenez-Andrade et al., 2010; Jimenez-Andrade et al., 2011; Kolosov et al., 2011; Kolosov et al., 2012; Muralidharan et al., 2013; Gdowski et al., 2017)
Colon	Colon-26	Bone	(Sabino et al., 2003)
Breast	4T1, 66.1, MADB-106, MRMT-1, walker 256, MAT B III, EO771, erlich	Hindpaw, vibrissal pad, bone, mammary pad	(Medhurst et al., 2002; Zhang et al., 2005; Liu et al., 2010; Yin et al., 2010; Wang et al., 2012; Calixto-Campos et al., 2013; Lofgren et al., 2018; Appel et al., 2019)
Lung	Lewis lung, ETCC clone 1,642	Bone, hindpaw, back, sciatic nerve	(Constantin et al., 2008; Isono et al., 2011; Maeda et al., 2016; Xiao et al., 2016; Wakabayashi et al., 2018)
Oral	SCC-158	Gingiva, hindpaw	(Nagamine et al., 2006; Shinoda et al., 2008; Hironaka et al., 2014)
Melanoma	B16-F10, B16-BL6	Bone, hindpaw	(Kuraishi et al., 2003; Fujita et al., 2010; Gao et al., 2015)
Sarcoma	MC57G, NCTC2472, MethA	Bone, sciatic nerve	(Schwei et al., 1999; Honore et al., 2000a; Honore et al., 2000b; Cain et al., 2001; Luger et al., 2001; Wacnik et al., 2001; Wacnik et al., 2003; King et al., 2007; Hald et al., 2009a; Hald et al., 2009b), (Halvorson et al., 2005a; Sevcik et al., 2005a; Wacnik et al., 2005a; Sevcik et al., 2005b; Wacnik et al., 2005b; Mouedden and Meert, 2005; Shimoyama et al., 2005), (Sabino et al., 2003)
Bladder	MBT-2	Bladder	(Roughan et al., 2004b; Roughan et al., 2014)
Myeloma	5TGM1-GFP	Systemic, bone	(Diaz-delCastillo et al., 2020)

To date, genetic cancer models have only been utilized to study pancreatic cancer pain [e.g., SV40 (Saenz Robles and Pipas, 2009), KPC (Biankin et al., 2012; Singhi et al., 2019)]. Genetically engineered models allow for the study of pain and neuroplasticity throughout the initiation and transformation of normal cells to malignancy as well as natural dissemination and metastasis. Typically, genetic models closely recapitulate human disease because they are based on specific genetic mutations that have been documented in patient populations. One of the greatest benefits of genetically engineered models is that the natural microenvironment remains intact, which permits modeling of complex processes that require interactions between multiple cells types (e.g., axonogenesis, metastasis, immune regulation). However, maintaining genetic models presents practical challenges. Several models have an average age of onset between 40 weeks and 20 months (Ding et al., 2016). Furthermore, as the number of transgenic alleles in a model increases, the cost-effectiveness and breeding efficiency decrease, making them expensive and time-consuming. Thus, while genetic models can play an important part in understanding biological mechanisms and pharmacology, they may not be ideal for high throughput testing (Webster et al., 2020).

Chemical-induced carcinogenesis has been utilized to study oral cancer pain (e.g., 4-nitroquinoline 1-oxide (4NQO) (Lam et al., 2012; Scheff et al., 2017; Scheff et al., 2018)) and colon cancer pain [e.g., azoxymethane (AOM) and dextran sulfate sodium (DSS) (Chartier et al., 2020)]. Like genetic models, chemical models allow for the study of pain development during the multistage dynamic carcinogenicity from initiation and progression. While some studies have employed local exposure (Bersch et al., 2009), a major benefit of these models is that the chemical is typically given systemically (e.g., drinking water) and can be used across multiple species. However, to date, cancer-related pain behavior has only been assessed in mouse and rat models. Additionally, due to uncontrolled exposure to the chemical, not all animals develop the same lesion at the same time, and a variety of lesions can be seen in a single rodent. While this can be clinically relevant as multiple primaries do occur in patients, high variability in the site and number of lesions between animals can also severely limit interpretations of pharmacology and behavioral studies. Unintended esophageal and gastrointestinal lesions are also possible and may confound results using spontaneous pain behavior assays.

The most common treatment-related cancer pain modeled in animals is CIPN, wherein chemotherapeutic agents (e.g., paclitaxel, oxaliplatin) are administered to animals resulting in dose-dependent damage to peripheral nervous system. The severity and temporal dynamics of neuropathy depend on the type of neurotoxic antineoplastic agent, dosage, and route of administration. CIPN rodent models have been extensively devised and studied in the last 30 years (Cavaletti et al., 2019). There is a wide variety of strains utilized, chemotherapeutic agents and routes of administration employed which have been systematically reviewed here (Gadgil et al., 2019). Many advantages are typical of these models: small size and prolific nature of the animals, ease of handling, along with the availability of reliable methods for peripheral nerve assessment. One of the most important benefits of this model is reproducibility within consistent methodology. Mouse CIPN models utilizing either paclitaxel or cisplatin have high efficacy in causing CIPN across sex and strains which is similar to clinical studies demonstrating a high incidence of CIPN in patients treated with these agents (Seretny et al., 2014; Molassiotis et al., 2019). However, one of the model’s major limitations is the high degree of variability regarding the chemotherapeutic agents used as well as the dose and route of administration chosen. Inconsistency in the model characteristics makes pharmacological conclusions across publications impossible to interpret.

### Cancer-Related Nociceptive Behavior Assays

Due to the variety of clinical presentations and comorbidities (e.g., affective components) in cancer-related pain, choosing among nociceptive assays remains challenging. A comprehensive list is compiled in Table 2. The most used behavioral assay for cancer pain is evoked mechanical sensitivity measured by quantification of responses to the application of standardized von Frey monofilaments to or near the site of inoculation (e.g., hindpaw, pancreas, vibrissal pad). One benefit of this assay is its common use in non-cancer pain literature. However, subjectivity limits interpretation of results. An animal’s lack of response to a noxious stimulus could indicate analgesia, paralysis, sedation, or lack of motivation; additional tests must screen for side effects that might confound the behavioral result. The alignment of animal research and clinical practice requires the use of similar behavioral endpoints. Hence, function-based tests, which facilitate the identification of drugs that inhibit nociception in the absence of disruptive side effects, are growing in popularity. Operant and function-related assays [e.g., gnawing (Dolan et al., 2010), wheel running (Tang et al., 2016), grid climbing (Falk et al., 2017)] have been used as an index of cancer-related pain; many of these behaviors and their clinical relevance have been thoroughly reviewed here (Tappe-Theodor et al., 2019).

**TABLE 2:** Cancer-related nociceptive behavior assays.

Pain type	Behavior	Pain assay	References
CIBP	Mechanical	Von frey	(King et al., 2007; King et al., 2008; Hald et al., 2009b; Ungard et al., 2014; Gdowski et al., 2017; Remeniuk et al., 2018)
	Thermal	Hargreaves, acetone, hotplate	(King et al., 2007; Miao et al., 2010)
	Spontaneous	CPP, open field activity, flinching, guarding, weight bearing, gait analysis, vocalizations, burrowing	(King et al., 2007; King et al., 2008; Ungard et al., 2014; Slosky et al., 2016; Gdowski et al., 2017; Remeniuk et al., 2018; Zhang et al., 2018; Buehlmann et al., 2019; Miladinovic et al., 2019; Sliepen et al., 2019)
	Function-induced	Forced limb use (limping, guarding), grip force, grid-climbing	—
	Breakthrough	Condition place aversion	(Havelin et al., 2017)
Oral cancer	Mechanical	Von frey	(Pickering et al., 2008; Ye et al., 2018; Salvo et al., 2020)
	Thermal	Hargreaves	(Ye et al., 2012; Ye et al., 2014; Salvo et al., 2020)
	Function-induced	Dolognawmeter	(Ye et al., 2011; Lam et al., 2012; Scheff et al., 2017; Scheff et al., 2018; Ye et al., 2018; Scheff et al., 2020)
	Spontaneous	CPP	(Chodroff et al., 2016; Scheff et al., 2019)
Pancreatic cancer	Mechanical	Von frey, visceromotor reflex	(Han et al., 2016; Wang et al., 2017)
	Spontaneous	Home cage activity, open field activity, hunching, vocalizations, grimace, wheel running, CPP	(Selvaraj et al., 2017; Wang et al., 2017)
CIPN	Mechanical	Von frey, randall-selitto	(Casals-Diaz et al., 2009; Han et al., 2018; Kyte et al., 2018; Laumet et al., 2019; Luo et al., 2019; Ma et al., 2019; Shahid et al., 2019; Bruna et al., 2020)
	Thermal	Hargreaves, hot plate, acetone, cold plantar test	(Chine et al., 2019; Luo et al., 2019; Shahid et al., 2019; Tonello et al., 2019; Bruna et al., 2020)
	Function-induced	Sensory and motor nerve conduction, functional autonomic tests, rota-rod, gait analysis	(Liu et al., 2018; Laumet et al., 2019; Luo et al., 2019; Bruna et al., 2020)
	Spontaneous	Burrowing, wheel running, CPP, adhesive recognition test	(Park et al., 2013; Flatters et al., 2017; Laumet et al., 2019; Toma et al., 2019; Bruna et al., 2020)
Colon cancer	Spontaneous	Burrowing, grimace	(Chartier et al., 2020)
Breast cancer	Mechanical	Von frey, hargreaves	(Lofgren et al., 2018)
	Spontaneous	Open field activity	(Lofgren et al., 2018)
Bladder cancer	Thermal	Hargreaves	(Roughan et al., 2014)
	Spontaneous	Open field, CPP, hunching, vocalization	(Roughan et al., 2004b; Roughan et al., 2014)

Spontaneous pain behavior is one of the most difficult components of cancer pain to manage and is therefore the most consistent end point against which the efficacy of a candidate drug is tested. The major limitation to using spontaneous pain as the end point is that the methodology differs substantially in the literature; spontaneous cancer-related pain has been measured indirectly using hunching (Lindsay et al., 2005a; Sevcik et al., 2006; Stopczynski et al., 2014; Wang et al., 2017; Kajiwara et al., 2020), open field activity (Stopczynski et al., 2014; Selvaraj et al., 2017; Hirth et al., 2020), home cage activity (Selvaraj et al., 2017; Hirth et al., 2020), voluntary wheel running (Selvaraj et al., 2017), vocalizations (Lindsay et al., 2005a; Sevcik et al., 2006), conditioned place preference (Selvaraj et al., 2017) as well as impressions of appearance [e.g., grimace scale (Kajiwara et al., 2020), coat condition (Roughan et al., 2004a)]. While many of these natural animal behaviors have minimal operator influence and can be translated to clinical representation of cancer-related pain, high variability of scoring criteria across studies and indirect output greatly limits the ability to assess the therapeutic impact. Additionally, distinguishing between spontaneous and “breakthrough” pain remains challenging, though recently an adaptation to the conditioned place aversion assay has been validated to measure movement-evoked breakthrough pain specifically (King et al., 2007; Havelin et al., 2017).

### Pharmacology From Bench to Bedside

Nerve growth factor (NGF) has been considered the most potent pain inducer across multiple cancer models and currently holds the most promise for management of pain associated with cancer initiation and progression. Inhibition of NGF binding to the receptor TrkA in preclinical models strongly reduced mechanical, thermal and spontaneous facets of cancer-induced bone pain (Halvorson et al., 2005b; Jimenez-Andrade et al., 2011; Buehlmann et al., 2019), pancreatic cancer pain (Stopczynski et al., 2014; Amit et al., 2019), and oral cancer pain (Ye et al., 2011). A phase II clinical trial using anti-NGF antibody, fulranumab, as adjunctive therapy for cancer-related pain found no significant effect on pain intensity via visual analog scale; however, significant improvement on the Brief Pain Inventory subscales suggested improved quality of life (Slatkin et al., 2019). Additionally, there are two ongoing clinical trials, one testing the analgesic efficacy of a TrkA inhibitor on cancer patients with solid tumors or lymphoma (phase I, NCT03556228) and the other measuring the efficacy of anti-NGF monoclonal antibody tanezumab in the treatment of cancer pain due to bone metastasis in patients already taking background opioid therapy (phase III, NCT02609828).

For treatment-related cancer pain, accumulating evidence indicates that the initiation and progression of CIPN are tightly related with chemotherapeutic agent-induced impairment of intraepidermal nerve fibers (IENF) (Koskinen et al., 2011), oxidative stress (Butturini et al., 2013), abnormal spontaneous discharge, ion channel activation (Zhang and Dougherty, 2014), up-regulation of various pro-inflammatory cytokines, and activation of the neuro-immune system (Sisignano et al., 2014; Makker et al., 2017). A phase III clinical trial using duloxetine, a serotonin and norepinephrine reuptake inhibitor, for treatment of pain associated with CIPN found that the use of duloxetine compared with placebo for 5 weeks resulted in a greater reduction in pain (NCT00489411). Calmangafodipir, mimicking the mitochondrial enzyme manganese superoxide dismutase, is currently involved in two ongoing clinical trials to establish the efficacy in prevention of chronic CIPN induced by oxaliplatin (phase III, NCT03654729 and NCT04034355).

## Considerations for Reverse Translation

Despite the large amount of human and experimental studies, no effective prophylactic treatment exists for cancer-related pain, and treatments (e.g., opioids) remain flawed. Identification of new targets for novel therapies to treat cancer-related pain requires models that better recapitulate interactions between cancer and its microenvironment, along with standardization of such assays and methodologies. Since so many promising studies fail in clinical translation, one must question the inherent translatability of the models themselves. While cancer-evoked nociceptive responses in animals echo some of the patients’ symptoms, the current models fail to address the complexity of interactions within the natural disease state. One of the major hypotheses for the etiology of focal cancer pain is cancer-secreted mediator-induced activation of the sensory nerve fibers innervating the cancer microenvironment (Jimenez Andrade and Mantyh, 2010; Schmidt, 2014; Lam, 2016). Therefore, anatomic site and neoplastic cell type should not be taken for granted when considering the translational relevance of the cancer pain model. Secreted mediators can differ depending on the cancer cell type (Sabino et al., 2003; Scheff et al., 2017; Scheff et al., 2020). Cancer-induced bone pain literature includes the most heterogeneity regarding cancer cell lines used (Currie et al., 2013). Substantial variability in pain behaviors and pharmacology may be attributed to the cancer cells selected to interact with peripheral nociceptive neurons; for this model multiple cancer cell lines might be required to determine if the findings are specific to one type of cancer or can be generalized to all bone metastasis. Similarly, variability in dosing and chemotherapeutic agent used in CIPN animal models could affect the consistency of findings (Gadgil et al., 2019).

To replicate symptoms observed in patients, reverse translation requires characterization of the pain associated with cancer or cancer treatment as either nociceptive, neuropathic, or mixed. Measures like numbness, tingling and ongoing pain rely on verbal report from the patient and often occur spontaneously. Fortunately, investigations into novel measures of ongoing pain in rodents are emerging (Tappe-Theodor and Kuner, 2014). A combination of pain assays including spontaneous pain should be used to demonstrate the translation of the cancer pain model to the clinical representation. However, consistency in criteria to score spontaneous pain across models is needed. Additionally, the stage of cancer progression at which pain develops in the animal model should align with the clinical representation. For example, oral squamous cell carcinoma pain in patients is thought to develop during the transition from precancerous lesion to malignancy (Lam and Schmidt, 2011). The 4NQO carcinogen model appears to be clinically representative with function-induced nociceptive behavior initiating at early stages in tumorigenesis (Scheff et al., 2017; Scheff et al., 2018); however, nociceptive behavior in an orthotopic xenograft mouse model does not develop for up to 14 days after inoculation (Ye et al., 2018; Scheff et al., 2019) suggesting that this model is more appropriate for pharmacological approaches to treat cancer-related pain at later stages in the disease when tumor burden is an active component. Lastly, the impact of age (Fujii, 1991; Lindsay et al., 2005b; Oh et al., 2018), sex (Scheff et al., 2018; Scheff et al., 2019; Rubin et al., 2020) and rodent strain (Vermeirsch and Meert, 2004; Zhang and Lao, 2012; Ono et al., 2015) can greatly impact both tumor development and nociceptive behavior and should be taken into consideration when designing a study. The cancer-related pain field needs to work together to standardize the methodology regarding both animal models and pain assays to increase the potential for reproducibility and clinical translation.

The cancer biology field is rapidly growing, and advances in cancer detection and therapy have improved patient prognosis. Preclinical models of several cancers [e.g., oral (Li et al., 2020), pancreas (Yin et al., 2015; Bisht and Feldmann, 2019), breast (Whittle et al., 2015; Holen et al., 2017)] have been extensively studied to determine a suitable research animal model that reflects the intricacies of cancer biology. In order to fully match the achievements in the cancer field broadly and understand the pain that may develop prior to detection or in response to treatments, we need to integrate the animal models most commonly used in cancer biology into the cancer-related pain field. Assimilation and standardization across both fields will allow for better translational findings across preclinical and clinical modalities. For example, patient-derived xenograft models (Aparicio et al., 2015) present a potential opportunity for patient-reported pain to be recapitulated in a transplantation mouse model with preservation of the genotypic and phenotypic diversity of the original tumor tissue. It is also imperative to consider facets of tumor biology beyond the nociceptive system (i.e., tumor growth, immune response) in pharmacological studies related to cancer pain; novel cancer pain therapies should not exacerbate cancer progression and interpretation of analgesia should be considered along with tumor size. Lastly, reverse translation could also be improved through the inclusion of large animal models [i.e., porcine (Robertson et al., 2020), canine (Kamano et al., 1988)]. There have been significant advances in veterinary oncology as well as validation of pain scales for companion animals (Brown et al., 2015; Lascelles et al., 2019) which provides an opportunity to study spontaneous cancer pain in larger species (Brown et al., 2015; Brown, 2016; Monteiro et al., 2018). To date, the cancer-related pain field has yet to integrate standardized pain assessment instruments for large animals (Henze and Urban, 2010; Viscardi et al., 2017; Lascelles et al., 2019; Luna et al., 2020).

## Conclusion

All cancer-related pain models have advantages and disadvantages, and there is no ideal cancer-related pain model that will perfectly recapitulate the human experience. The appropriate model depends on the cancer-related pain condition and the specific methods used. Despite limitations, the field has begun to provide insight into the mechanisms that generate and maintain cancer-related pain while discovering potential therapeutic strategies to treat it. Additionally, there has been a recent surge in data suggesting that manipulation of neuronal activity by cancer cells may be a central mechanism for cancer progression (Faulkner et al., 2019; Zahalka and Frenette, 2020). Thus, targeting sensory neurons in the cancer microenvironment may be a potentially actionable therapeutic strategy to stop cancer pain as well as slow cancer growth. As this new field of cancer neurobiology emerges, full collaboration between cancer biologists, neurobiologists and immunologists is pivotal for success.

## Author Contributions

All authors, JP-F, JS, NS, drafted the work, contributed to work design, revised it, and approved the final version to be published and are accountable for all aspects of the work.

## Conflict of Interest

The authors declare that the research was conducted in the absence of any commercial or financial relationships that could be construed as a potential conflict of interest.
